# Synthesis, X-ray diffraction analysis, quantum chemical studies and *α*-amylase inhibition of probenecid derived *S*-alkylphthalimide-oxadiazole-benzenesulfonamide hybrids

**DOI:** 10.1080/14756366.2022.2078969

**Published:** 2022-05-26

**Authors:** Bilal Ahmad Khan, Syeda Shamila Hamdani, Muhammad Naeem Ahmed, Shahid Hameed, Muhammad Ashfaq, Ahmed M. Shawky, Mahmoud A. A. Ibrahim, Peter A. Sidhom

**Affiliations:** a Department of Chemistry, The University of Azad Jammu and Kashmir, Muzaffarabad, Pakistan; b Department of Chemistry, Quaid-i-Azam University, Islamabad, Pakistan; c Department of Physics, University of Sargodha, Sargodha, Pakistan; d Science and Technology Unit (STU), Umm Al-Qura University, Makkah, Saudi Arabia; e Computational Chemistry Laboratory, Chemistry Department, Faculty of Science, Minia University, Minia, Egypt; f Department of Pharmaceutical Chemistry, Faculty of Pharmacy, Tanta University, Tanta, Egypt

**Keywords:** Oxadiazole, probenecid, X-ray diffraction, enzyme inhibition, molecular modelling

## Abstract

Sulphonamide and 1,3,4-oxadiazole moieties are present as integral structural parts of many drugs and pharmaceuticals. Taking into account the significance of these moieties, we herein present the synthesis, single-crystal X-ray analysis, DFT studies, and *α*-amylase inhibition of probenecid derived two *S*-alkylphthalimide-oxadiazole-benzenesulfonamide hybrids. The synthesis has been accomplished in high yields. The final structures of both hybrids have been established completely with the help of different spectro-analytical techniques, including NMR, FTIR, HR-MS, and single-crystal X-ray diffraction analyses. In an effort to confirm the experimental findings, versatile quantum mechanical calculations and Hirshfeld Surface analysis have been performed. *α*-Amylase inhibition assay has been executed to investigate the enzyme inhibitory potential of both hybrids. The low IC_50_ value (76.92 ± 0.19 μg/mL) of hybrid **2** shows the good *α*-amylase inhibition potential of the respective compound. Ultimately, the binding affinities and features of the two hybrids are elucidated utilising a molecular docking technique against the *α*-amylase enzyme.

## 1. Introduction

Drug discovery is a continuous challenge and keeps fascinating the researchers worldwide continuously regardless of the time and efforts required. Heterocycles, specially oxadiazoles, have been very important in drug discovery due to their important role in medicines. The use and demand of oxadiazoles motifs in drugs are increasing continuously and can be seen from their increasing number of patents filed, up to increase in 100% in last 10 years from 2000 to 2008, still this number is increasing on^1^. Nowadays, a considerable amount of drugs in use possess oxadiazole moiety, a few examples are zibotentan^2–3^, used for curing cancer, raltegravir, an important antiretroviral drug against HIV, and ataluren, used for the treatment of cystic fibrosis^4^. Oxadiazole rings help in fulfilling the dream of drug discovery in multiple ways, acting as an important part of pharmacophore, which stimulates the binding of chromatophore to ligand^5^, as a linker to fix the proper position of the substituent in space^6^ and help in controlling the molecular properties^7^. The importance of oxadiazole is not limited to the medicinal field but is also equally important in the industrial zone as a thermal stabiliser for polymer synthesis and has found wide applications in optics^8–10^. Many discoveries have proved oxadiazole motif has a broad range of pharmaceutical importance as an antidiabetic^11^, lipoxygenase inhibitor^12^, anti-inflammation^13^, anti-infection^14^, elastase inhibitors^15^, amylase inhibitor^16^, antibacterial^17^, antiobesity^18^, nonpeptidic procollagen C-proteinase inhibition^19^, anticancer^20^, and dipeptidyl peptidase IV inhibition^21^. Moreover, oxadiazoles are also reported to inhibit monoamine oxidase^22^, niacin receptor (GPR109A) agonist^23^, larvicide^24^, antifungal^25^, and glutaminyl cyclase inhibitors^26^.

Probenecid has been an important drug since the nineteenth century and is used in the treatment of multiple diseases, mainly as a uricosuric agent to fight hyperuricaemia. Probenecid plays a vital role in active brain transportation^27^, curing gout^28^, as a human carbonic anhydrase inhibitor^29^, and plays a significant role as an assistant to enhance blood vessels^27^.

*α*-Amylase (ptyalin) is a digestive enzyme found to an ample extent in animals and humans. It is produced by salivary glands and pancreas at a neutral to mildly acidic pH. It behaves as a catalyst in the hydrolysis of complex polysaccharides (starch and glycogen) into shorter chains that ultimately produce glucose, thus facilitating digestion. Excessive *α*-amylase in blood accelerates starch breakdown resulting high concentration of sugars. Controlling the *α*-amylase in the body can result in the reduction of hyperglycaemia and obesity. *α*-Amylase is a good target for developing inhibitors for the treatment of widely spread diabetes and obesity^30–32^.

Acarbose is a commercially available drug prescribed to manage diabetes mellitus type II by inhibiting the salivary and pancreatic *α*-amylase and *α*-glucosidase. However, it is decomposed by bacterial *α*-amylases in the intestine. Acarbose inhibits excessive *α*-amylase, thus retarding the hydrolysis of polysaccharides and controlling the blood sugar levels.

**Figure 1.**
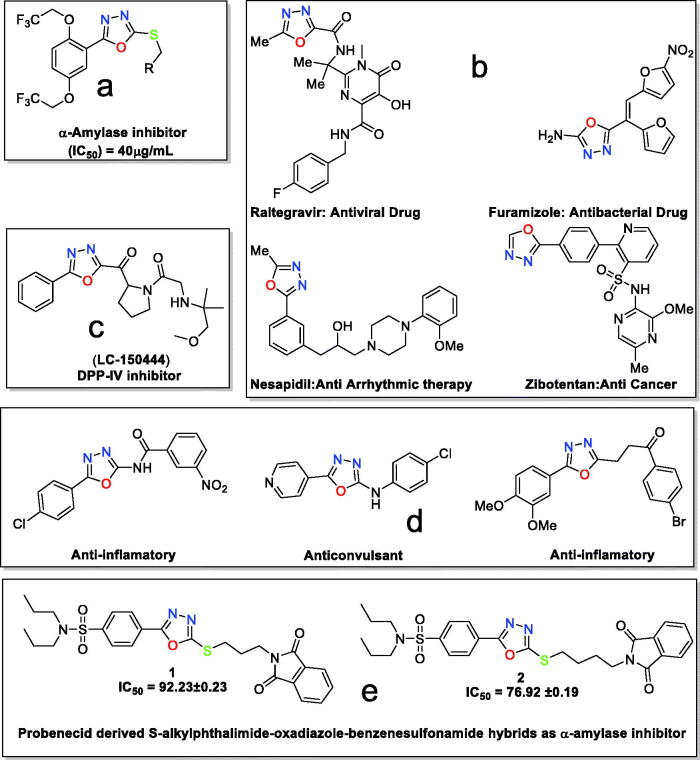
(a) 1,3,4-Oxadizole moiety containing compound as *α*-amylase inhibitor; (b) 1,3,4-oxadiazole moiety in drugs; (c) 1,3,4-oxadizole moiety containing compound in preclinical trials; (d) 1,3,4-oxdiazole displaying various biological activities; and (e) present work.

1,3,4-Oxadiazoles have been synthesised in great structural diversity and studied for their inhibition potential against the *α*-amylase enzyme. A brief literature survey showed that a series of 5–(2,5-bis(2,2,2-trifluoroethoxy)phenyl)-1,3,4-oxadiazole-2-thiol derivatives had inhibited *α*-amylase in the range of IC_50_=40.00–80.00 μg/mL (Figure 1(a))^3^. l-2-(*β*-D-glucopyranosyl)-1,3,4-oxadiazoles have been evaluated for their antidiabetic potential^34^. Similarly, *S*-benzyl substituted 1,3,4-oxadiazole-2-thiol derivatives have been found to inhibit the *α*-amylase enzyme in the 100 μM concentration^16^.

Besides *α*-amylase inhibition, 1,3,4-oxadizole-based compounds have been found in commercially available drugs such as raltegravir as an antiviral agent, furamizole as an antibacterial agent, nesapidil as an antiarrhythmic agent and tiodazosin as an antihypertensive agent^35^. The structures of commercial drugs are shown in (Figure 1(b)). 1,3,4-Oxadiazole containing compound LC-150444, disclosed in (Figure 1(c)), is in preclinical trials for the inhibition of DPP-IV^36^.

1,3,4-Oxadiazoles have become a pharmacophore possessing a variety of biological potentials, including anticonvulsant^37^, anti-inflammatory^38^, antitubercular activity^39^, and many others^35^ (Figure 1(d)).

Towards discovering new compounds for enzyme inhibition and keeping in loop our previous work^40–41^, herein we are reporting the synthesis, crystallographic analysis, DFT investigation, and *α*-amylase inhibition of two hybrid compounds (Figure 1(e)). The two hybrids are designed to be a combination of probenecid and oxadiazole moieties and are expected to have high biological activities.

## 2. Materials and methods

### 2.1. Experimental

Starting materials, reagents and solvents are procured from Merk and Sigma Aldrich and utilised directly without any further treatment. Melting points of both hybrids are recorded by using the Gallenkamp melting point apparatus (MP-D) and are reported as uncorrected. NMR spectra (^1^H and ^13 ^C) are recorded on an AV-300 spectrometer (300 MHz) manufactured by Bruker. FT-IR spectra of both hybrids are recorded in ATR (Attenuated Total Reflectance) mode by using the Fourier Transform Infra-Red spectrophotometer supplied by Shimadzu.

### 2.2. Synthesis

#### 2.2.1. Preparation of probenecid derived S-alkylphthalimide-oxadiazole-benzenesulfonamide hybrids (1 and 2)

The probenecid (20 mmol) is taken in 30 mL of methanol and 0.5 mL of conc. sulphuric acid is added and stirred at reflux for 12 h. Then concentrated and washed with 150 mL of a saturated aqueous solution of sodium bicarbonate and extracted three times with ethyl acetate (3 × 50 mL). The organic layer is dried over anhydrous sodium sulphate and concentrated to have pure probenecid ester. From probenecid ester, the probenecid hydrazide is synthesised by adopting the following modified procedure from the literature^42–44^. The solution of probenecid ester (15 mmol) in 30 mL of methanol is taken in a round bottom flask, and hydrazine hydrate (80%, 0.06 mol) is dropped slowly into it. The reaction mixture is refluxed for 8 h. Upon completion of the reaction, ice cold water is added to the reaction mixture at room temperature. The precipitated crude probenecid hydrazide attained is filtered, dried, and recrystallized from methanol.

In the next step, the probenecid hydrazide (10 mmol) is dissolved in methanol containing potassium hydroxide (30 mmol) at room temperature. After a few minutes of stirring, carbon disulphide (0.06 mol) is dropped into the reaction mixture and further refluxed for 12 h. It is concentrated to less than half, poured into the ice-cold water, and acidified with 1 N HCl to pH = 2. The resultant crude precipitates are then cleaned with warm water and recrystallized from methanol to have pure oxadiazole-2-thiol.

To a solution of the corresponding oxadiazole (1 mmol) and bromo-alkyl-substituted phthalimide (1 mmol) in 15 mL acetone, 1 equivalent of potassium phosphate is added and stirred further at room temperature for 4 h. It is concentrated and recrystallized from methanol to obtain pure *S*-alkylphthalimide-oxadiazole-benzenesulfonamide hybrids 1 and2 in 79% and 83% yields, respectively.

##### 2.2.1.1. *4–(5-(3–(1,3-Dioxoisoindolin-2-yl)propylthio)-1,3,4-oxadiazol-2-yl)-N,N-dipropylbenzenesulfonamide* (1)


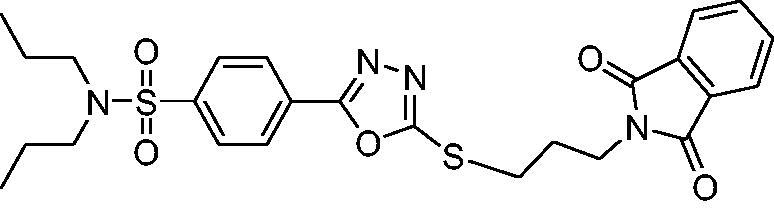


Colourless solid; %age yield: 79; Retention factor (R*_f_)_:_* = 0.45 (CHCl_3_: CH_3_COCH_3_, 9:1); melting point; 157–158 °C; ^1^H-NMR (300 MHz, CDCl_3_): *δ* (ppm) 0.87 (t, *J* = 7.35 Hz, 6 H, –CH_3_) 1.45–1.66 (m, 4 H, CH_3_–CH_2_–) 2.23–2.32 (m, 2 H, CH_2_CH_2_CH_2_) 3.02–3.19 (m, 4 H, N(CH_2_)_2_) 3.36 (t, *J* = 7.16 Hz, 2 H, S-CH_2_) 3.89 (t, *J* = 6.59 Hz, 2 H, N–CH_2_) 7.70–7.77 (m, 2 H, Ar-H) 7.82–7.88 (m, 2 H, Ar–H) 7.88–7.96 (m, 2 H, Ar–H) 8.07–8.18 (m, 2 H, Ar-H); ^13 ^C NMR (75 MHz, CDCl_3_): *δ* (ppm) 11.12, 21.86, 28.42, 29.91, 36.42, 49.81, 76.57, 77.42, 123.35, 126.83, 127.10, 127.63, 131.87, 134.11, 142.93, 164.48, 165.11, 168.34; FT-IR ν(cm^−1^): 3025 (C–H, SP^2^), 2937 (C–H, SP^3^), 1708, 1620 (Carbonyl group), 1545 (C = N), 1337, 1151 (SO_2_ group); High Resolution Mass Spectrum (EI-TOF) [M^+^] Calcd. for: C_25_H_28_N_4_O_5_S_2_: 528.1501; found; 528.1500.

##### 2.2.1.2. 4–(5-(4–(1,3-Dioxoisoindolin-2-yl)butylthio)-1,3,4-oxadiazol-2-yl)-N,N-dipropylbenzenesulfonamide (2)


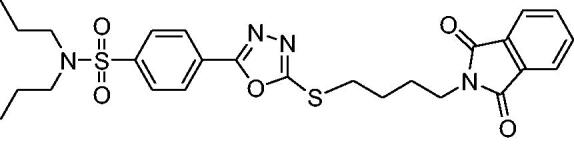


Colourless solid; %age yield: 83; Retention factor (*R_f_)*: = 0.51 (CHCl_3_: CH_3_COCH_3_, 9:1); melting point; 117–119 °C; ^1^H-NMR (300 MHz, CDCl_3_): *δ* (ppm) 0.88 (t, *J* = 7.35 Hz, 6 H, CH_3_) 1.47–1.63 (m, 4 H, CH_3_–CH_2_–) 1.91 (m, 4 H, CH_2_CH_2_CH_2_) 3.05 − 3.18 (m, 4 H, N(CH_2_)_2_) 3.37 (t, *J* = 7.16 Hz, 2 H, S–CH_2_) 3.75 (t, *J* = 6.59 Hz, 2 H, N–CH_2_) 7.68 − 7.76 (m, 2 H, Ar–H) 7.80 − 7.88 (m, 2 H, Ar–H) 7.90 − 7.96 (m, 2 H, Ar-H) 8.08–8.16 (m, 2 H, Ar–H); ^13 ^C-NMR (75 MHz, CDCl_3_): *δ* (ppm) 11.56, 22.30, 26.90, 27.90, 32.37, 37.52, 50.26, 77.43, 77.85, 123.68, 127.32, 127.53, 128.08, 132.38, 134.43, 143.37, 164.84, 168.76; FT-IR (cm^−1^): 3088 (C–H, SP^2^), 2970 (C–H, SP^3^), 1703, 1617 (Carbonyl group), 1543(C = N), 1332, 1160(SO_2_); High Resolution Mass Spectrum (EI-TOF) [M^+^] Calcd. for: C_26_H_30_N_4_O_5_S_2_: 542.1658; found; 542.1656.

### 2.3. Crystal structure determination

*X-ray diffraction data is collected on* Super Nova, Single source at offset, Atlas diffractometer with CuKα x-rays source (*λ* = 1.54184 Å). Multi-scan *CrysAlis PRO* 1.171.38.46 (Rigaku Oxford Diffraction, 2015) is used for empirical absorption correction spherical harmonics, implemented in SCALE3 ABSPACK. The structure solution is performed by SHELXT-2014^45^, whereas SHELXL 2019/2^46^ is employed for the refinement. All the atoms other than hydrogen are refined by assigning anisotropic displacement parameters. Isotropic displacement parameters are assigned to hydrogen atoms and are refined or placed by using the riding model. ORTEP-3^47^, PLATON^48^ and MERCURY 4.0^49^ software are used for graphical purposes. Files of both hybrids **1** and **2** have been assigned CCDC numbers 1963535 and 1963536, respectively, and can be obtained free of charge on-demand to CCDC 12 Union Road, Cambridge CB21 EZ, UK. (Fax: (+44) 1223 336–033: data_request@ccdc.cam.ac.uk).

### 2.4. Computational methodology

To verify the experimental results, versatile quantum mechanical calculations are carried out on the two hybrids (**1** and **2**). The studied hybrids are first optimised at the B3LYP/6-31G* level of theory^50–51^. Upon the optimised geometries, the vibrational frequency and single-point energy calculations are executed. Molecular electrostatic potential (MEP) maps are then generated using a 0.002 au electron density envelope according to the previous recommendations^52^. The highest occupied molecular orbital (HOMO) and lowest unoccupied molecular orbital (LUMO) distributions are also visualised for the optimised geometries. Moreover, the quantum theory of atoms in molecules (QTAIM)^53^ along with noncovalent interaction (NCI) index^54^ are utilised to identify the origin of interactions within the dimeric form of the studied hybrids based on the crystallographic coordinates. The QTAIM and NCI calculations are executed using Multiwfn 3.7 package^55^ and are graphed using Visual Molecular Dynamics (VMD) software^56^. All the adopted quantum mechanical calculations are performed at the B3LYP/6-31G* level of theory with the help of Gaussian 09 software ^57^.

### 2.5. Hirshfeld surface (HS) analysis

Hirshfeld surface (HS) analysis^58^ is executed to give an in-depth qualitative insight into the role of the main intermolecular interactions. Within the context of HS analysis, the normalised contact distance (*d*_norm_) surface along with its corresponding two-dimensional (2 D) fingerprint plots is generated to allow the identification of the crucial regions in the crystal packing of the investigated hybrids. The *d*_norm_ surfaces are mapped over a fixed colour scale ranging from red (–0.05 au) to blue (+0.75 au). The fingerprint plots are figured out using the translated 1.0 − 2.8 Å range, and reciprocal contacts are considered. As well, the shape index and curvedness properties are mapped with the colour range of −1.0 au (concave) to 1.0 au (convex) and range of −4.0 au (flat) to 0.40 au (singular), respectively. The generated Hirshfeld surfaces and the associated 2 D fingerprint plots are extracted using the CrystalExplorer17 software^59^.

### 2.6. Molecular docking

The technical specifics of the utilised molecular docking computations are elucidated in Ref.^60–64^. In concise, the resolved three-dimensional (3 D) crystal structure of *α*-amylase (PDB ID: 1OSE^65^) in complex with acarbose is downloaded from RCSB PDB and opted for docking computations. All crystallographic waters, ligand, and ions are removed. The H++ server is utilised to determine the protonation states of *α*-amylase amino acids ^66^. As well, all missing hydrogen atoms are inserted. The 3D structures of the two hybrids are generated utilising Omega2 software^67–68^. Merck Molecular Force Field 94 (MMFF94S), with the help of SZYBKI software^69–70^, is applied to minimise the 3D structures.

In this work, AutoDock4.2.6 software is employed to perform all molecular docking calculations^71^. The MGL tools (version 1.5.7) is utilised to create the pdbqt file for the *α*-amylase based on the AutoDock protocol^72^. The population size and the number of generations for molecular docking computations are adjusted to 300 and 27 000, respectively. Besides, the maximum number of energy evaluations (*eval*) is 25 000 000, while the genetic algorithm (*GA*) variable is set to 250. The remaining docking settings are left at their default values. The grid box size is adjusted to 60 Å × 60 Å × 60 Å, which is able to encompass the active site of *α*-amylase. AutoGrid4.2.6 program is adopted to create grid map files with a spacing value of 0.375 Å. The grid centre is located at the coordinates *X* = 32.644, *Y* = 38.464, and *Z* = −3.166. The root mean square deviation of 1.0 Å is utilised to cluster conformations, and the docking score is adopted to rank them ^73^. Additionally, the lowest docking score inside the largest cluster is deemed for adopting as a representative mode.

### 2.7. Enzyme inhibition activity

*α*-Amylase inhibitory activity of probenecid derived *S*-alkylphthalimide-oxadiazole-benzenesulfonamide hybrids (**1** and **2**) is achieved using the 3,5-dinitrosalicylic acid (DNS) method [41]. Compound solutions are prepared by dissolving each compound in dimethylsulphoxide (DMSO) to give a concentration of 1 mgmL^−1^. A measured volume from each compound solution is mixed separately with 200 μL of *α*-amylase solution (1 mgmL^−1^, pH 7.0) in a test tube and incubated for 10 min at 30 °C. 200 μL of the starch solution (1% in water (w/v)) is added to the tube and incubated for 3 min. 200 μl of DNS reagent (20 mL of 96 mM DNS and 12 g of sodium potassium tartrate tetrahydrate in 8.0 mL of 2 M NaOH) is added to this mixture for reaction termination and the mixture is kept in a boiling water bath for 10 min. After cooling the mixture to ambient temperature, 5 mL of distilled water is added to dilute it, and the absorbance is recorded at 540 nm using a UV-Visible spectrophotometer (Shimadzu UV 1800). A blank reaction mixture is also prepared to remove the background absorbance produced by each compound at various studied concentrations containing all other components except the enzyme solution. Acarbose (250–10 μgmL^−1^) is used as a potent amylase inhibitor to compare the inhibition potential of compounds (1–3), whereas the control incubation, representing maximum enzyme activity, is carried out by replacing the compound with 2% DMSO. All the tests are performed in triplicate. The *α*-amylase inhibitory activity is calculated as percent inhibition using the following formula:

$$\%\;\mathrm{\alpha -amylase}\;\mathrm{inhibition=}\frac{A_{o}-A_{i}}{A_{o}}\mathrm{\times 100}$$

where Ao is the absorbance of the mixture containing enzyme, starch, and DNS; Ai is the absorbance of the mixture containing enzyme, inhibitor (compound/acarbose), starch, and DNS. IC_50_ values (μgmL^−1^) are obtained from the graph plotted for % α-amylase inhibition obtained at five different concentrations (10–200 μgmL^−1^).

## 3. Results and discussion

### 3.1. Chemistry

**Scheme 1.**
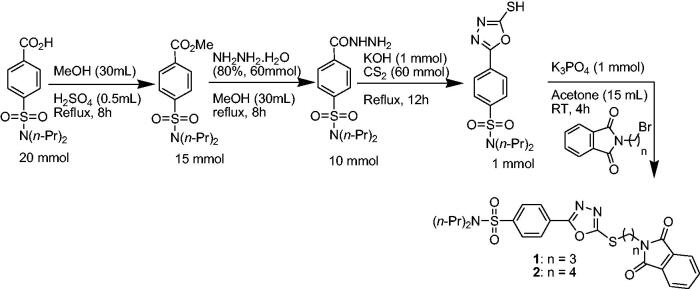
Synthetic approach for probenecid derived three *S*-alkylphthalimide-oxadiazole-benzenesulfonamide hybrids (**1** and **2**).

The synthetic approach adapted to access probenecid-derived *S*-alkylphthalimide-oxadiazole-benzenesulfonamide hybrids (**1** and **2**) during this work is shown in Scheme 1. The structures of both hybrids are established on the basis of FTIR, ^1^H NMR ^13^C NMR and HR-MS spectroscopies, and single-crystal X-ray crystallography.

In ^1^H NMR spectra of probenecid derived *S*-alkylphthalimide-oxadiazole-benzenesulfonamide hybrids (**1** and **2**), all the eight aromatic protons are found in the range of *δ* 7.68–8.25 ppm. A multiplet for four protons at *δ* 3.00–3.22 ppm is assigned to *N*-(CH_2_)_2_ in both compounds. The four protons of the other two methylenes of the *n*-isopropyl group are observed as a multiplet at *δ* 1.45–1.66 ppm in **1** and **2**. A triplet for six protons of two methyl groups on the *n*-isopropyl group is found between *δ* 0.87–0.89 ppm. A triplet for two protons at *δ* 3.36 and δ 3.37 ppm is assigned to each *S*-CH_2_ group in compounds **1** and **2**, respectively. The triplets for two protons at *δ* 3.89 ppm and 3.75 ppm are assigned to the *N*–CH_2_ group in compounds **1** and **2**, respectively. A multiplet for two protons at *δ* 2.23–2.32 ppm is assigned to the methylene group in the middle of the *n*-propyl chain in compound **1** whereas, a doublet of a doublet having coupling constant 4.05 and 1.98 Hz for four protons at *δ* 1.92 ppm is assigned to two methylene groups in the middle of the *n*-butyl chain.

In ^13^C NMR spectroscopic studies of compounds **1** and **2**, the two carbonyl carbons of phthalimide skeleton are observed at *δ* 168.34 and 168.76 ppm for compounds **1** and **2**, respectively. Similarly, the two carbon atoms present in the oxadiazole part of the hybrid are found at 165.18, 164.55 and 165.34, 164.47 ppm in hybrid **1** and **2**, respectively. The carbon atoms in the aromatic rings are found between *δ* 144–122 ppm. The shift between *δ* 49.80 and 50 ppm is assigned to N(CH_2_)_2_ of the *n*-isopropyl group in both hybrids. The shift at 36.49 and 37.14 ppm are assigned to *S*–CH_2_*N* and *N*–CH_2_ in hybrids **1** and **2**, respectively. The chemical shift at *δ* 29.98 and 31.99 ppm is assigned to *S*–CH_2_ in hybrids **1** and **2**, respectively. Other carbon atoms of the alkyl chains are observed below *δ* 30 ppm.

### 3.2. Molecular structures

**Figure 2.**
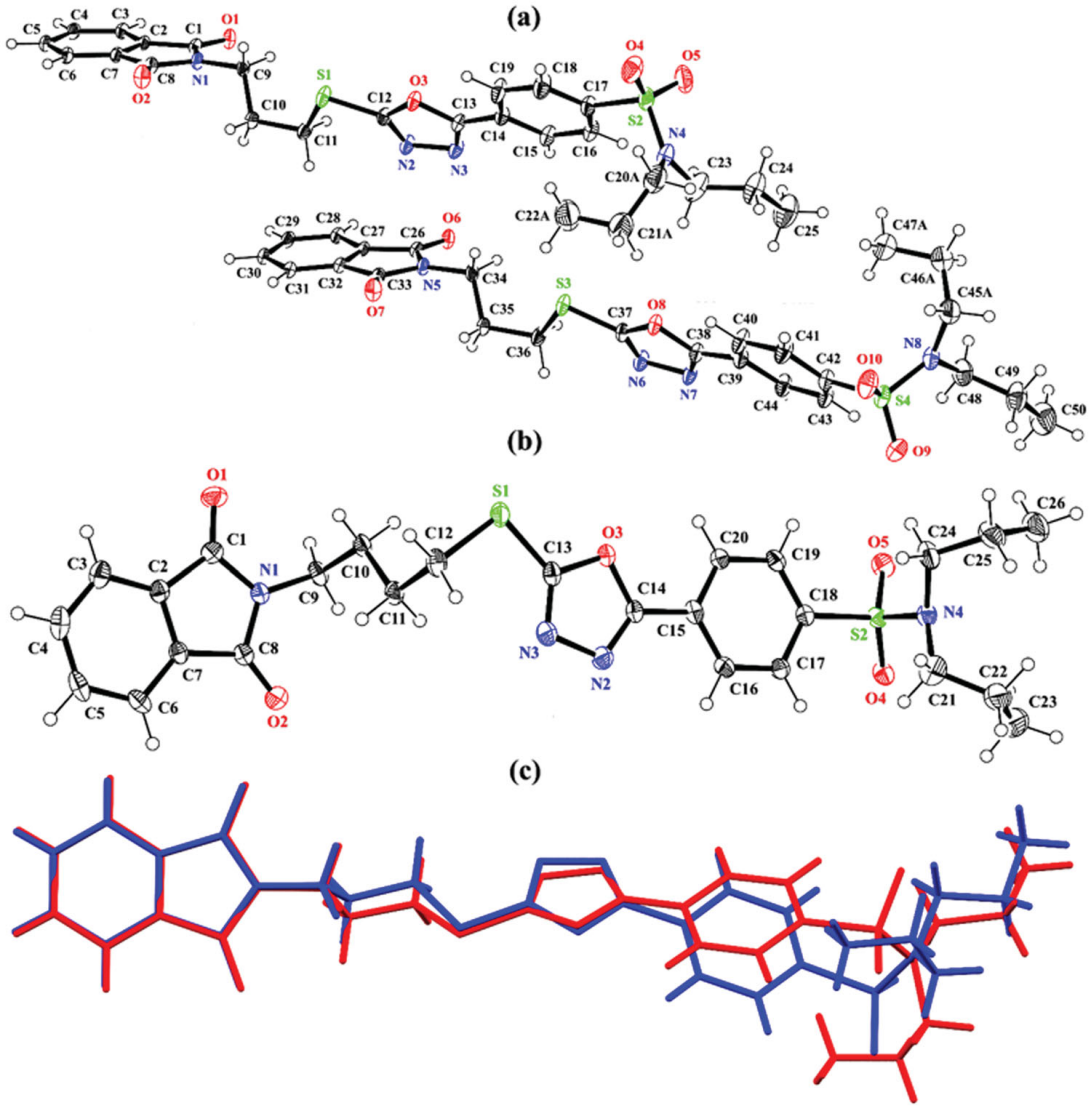
*ORTEP* diagram of hybrids **(a) 1** and **(b) 2** that are drawn at a probability level of 40%. Hydrogen atoms are shown by small circles of arbitrary radii. **(c)** Molecular overlay plot of hybrid **1**, molecule I (red) and molecule II (blue). The major of the disordered propyl groups in hybrid **1** are shown for clarity.

The experimental details of both hybrids **1** and **2** are given in Table 1. Both crystal structures are crystallised in a triclinic crystal system with space group P$\bar{1}.$ The asymmetric unit of hybrid **1** (Figure 2(a)) consists of two molecules (named molecule **I** and molecule **II**) that are different geometrically with respect to each other. In both molecules, molecule **I** (C1–C25/N1–N4/O1-O5/S1/S2) and molecule **II** (C26–C50/N5–N8/O6-O10/S3/S4), one of the propyl groups that is attached to N-atom is disordered over two sets of sites with an occupancy ratio of 0.666 (4): 0.334 (4). The disordered is solved by using EADP, SIMU, DELU, DFIX, and DANG restraints. In molecule **I**, the isoindoline-1,3-dione ring A (C1–C8/N1/O1/O2), 1,3,4-oxadiazole ring B (C12/C13/N2/N3/O3), and phenyl ring C (C14–C19) are planar with root mean square deviation of 0.0170, 0.0027 and 0.0071 Å, respectively with dihedral angles A/B and B/C is 51.9(6)° and 8.83(8)°, respectively. In molecule **II**, the isoindoline-1,3-dione ring D (C26–C33/N3/O6/O7), 1,3,4-oxadiazole ring E (C37/C38/N6/N7/O8), and phenyl ring F (C39–C44) are planar with root mean square deviation of 0.0227, 0.0027 and 0.0098 Å, respectively, with dihedral angles D/E and E/F is 54.42(6)° and 11.43(8)°, respectively. In order to further explore the difference between molecules **I** and **II**, a molecular overlay plot is formed by using Mercury 4.0 (Figure 2(c)). The molecules are inverted and then made to overlap. Molecules **I** and **II** are shown in red and blue colour, respectively. The root mean square deviation (RMSD) and the maximum deviation (Max. D) between molecules **I** and **II** are 1.6359 and 4.7768 Å, respectively. The major difference between molecules is in the orientation of the disordered propyl groups (Figure 2(c)).

**Table 1.** X-ray Parameters of both hybrids **1** and **2**.

Crystal parameters	1	2
Chemical formula	C_25_H_28_N_4_O_5_S_2_	C_26_H_30_N_4_O_5_S_2_
CCDC	1963535	1963536
*M*_r_	528.63	542.66
Crystal system, space group	Triclinic, *P*$\bar{1}$	Triclinic, *P*$\bar{1}$
Temperature (K)	150	150
*a*, *b*, *c* (Å)	7.6816 (3), 15.6060 (6), 21.4957 (8)	9.8274 (6), 10.7739 (7), 13.0693 (8)
*α*, *β*, *γ* (°)	73.111 (3), 83.817 (3), 89.441 (3)	101.706 (5), 106.908 (6), 93.204 (5)
*V* (Å^3^)	2450.75 (16)	1286.63 (14)
*Z*	4	2
Radiation type	Cu *K*α	Cu *K*α
µ (mm^–1^)	2.353	2.255
Crystal size (mm)	0.32 × 0.24 × 0.10	0.51 × 0.23 × 0.03
Diffractometer	Super Nova, Single source at offset, Atlas	Super Nova, Single source at offset, Atlas
Absorption correction	Multi-scan *CrysAlis PRO* 1.171.38.46 (Rigaku Oxford Diffraction, 2015) Empirical absorption correction using spherical harmonics, implemented in SCALE3 ABSPACK scaling algorithm.	Multi-scan *CrysAlis PRO* 1.171.38.46 (Rigaku Oxford Diffraction, 2015) Empirical absorption correction using spherical harmonics, implemented in SCALE3 ABSPACK scaling algorithm.
*T*_min_, *T*_max_	0.516, 0.795	0.394, 0.938
No. of measured, independent and observed [*I* > 2σ(*I*)] reflections	17120, 9497, 7809	4619, 4619, 3884
*R*_int_	0.028	0.040
(sin *θ*/λ)_max_ (Å^–1^)	0.617	0.599
*R*[*F*^2^ > 2σ(*F*^2^)], *wR*(*F*^2^), *S*	0.056, 0.160, 1.03	0.074, 0.217, 1.09
No. of reflections	9497	4619
No. of parameters	670	337
No. of restraints	166	–
H-atom treatment	H-atom parameters constrained	H-atom parameters constrained
Δ*ρ*_max_, Δ*ρ*_min_ (e Å^–3^)	0.77, −0.64	0.67, −0.51

In hybrid **2** (Figure 2(b)), the twined data is solved, and good values of the R-factors are acquired. The BASF value is found to be 0.35899. The isoindoline-1,3-dione ring A (C1–C8/N1/O1/O2), 1,3,4-oxadiazole ring B (C13/C14/N2/N3/O3), and phenyl ring C (C15–C20) are planar with root mean square deviation of 0.0213, 0.0031 and 0.0066 Å, respectively with dihedral angles A/B and B/C is 3.39(3)° and 5.7(3)°, respectively.

**Figure 3.**
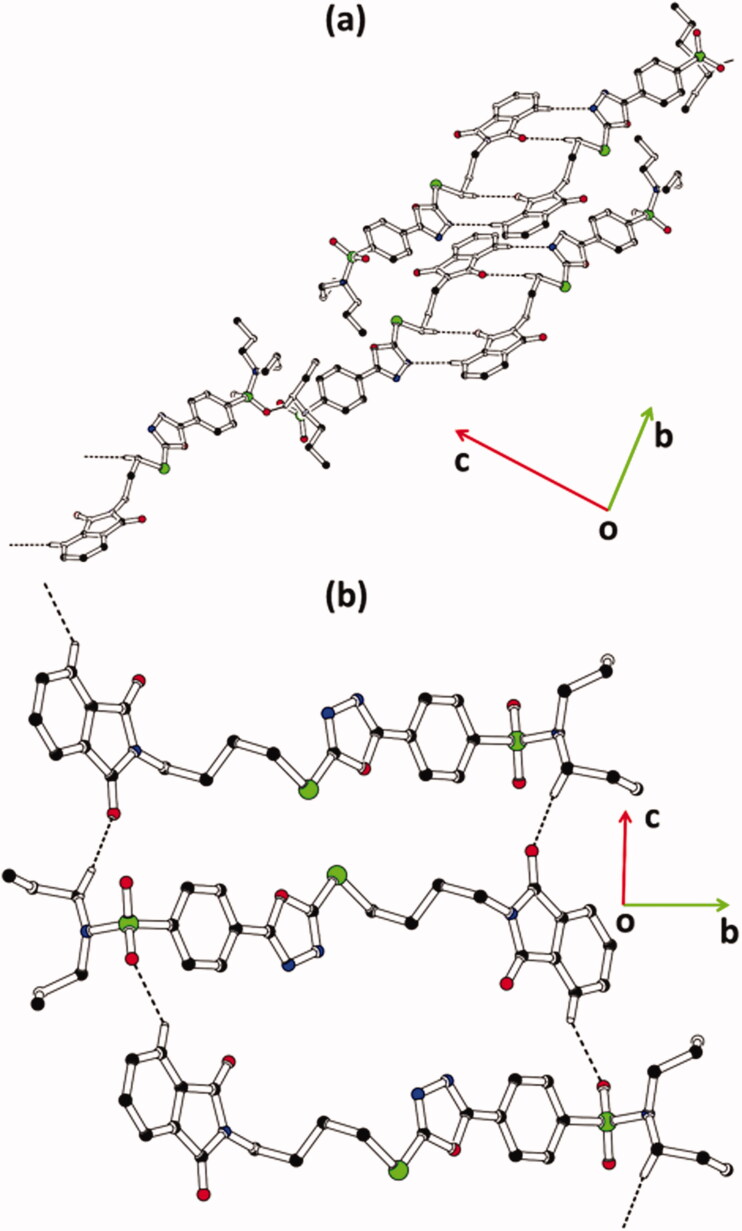
Packing diagram of hybrids **(a) 1**, and **(b) 2**. Selected hydrogen atoms are shown for clarity. The major of the disordered propyl groups in hybrid **1** are shown for clarity.

**Figure 4.**
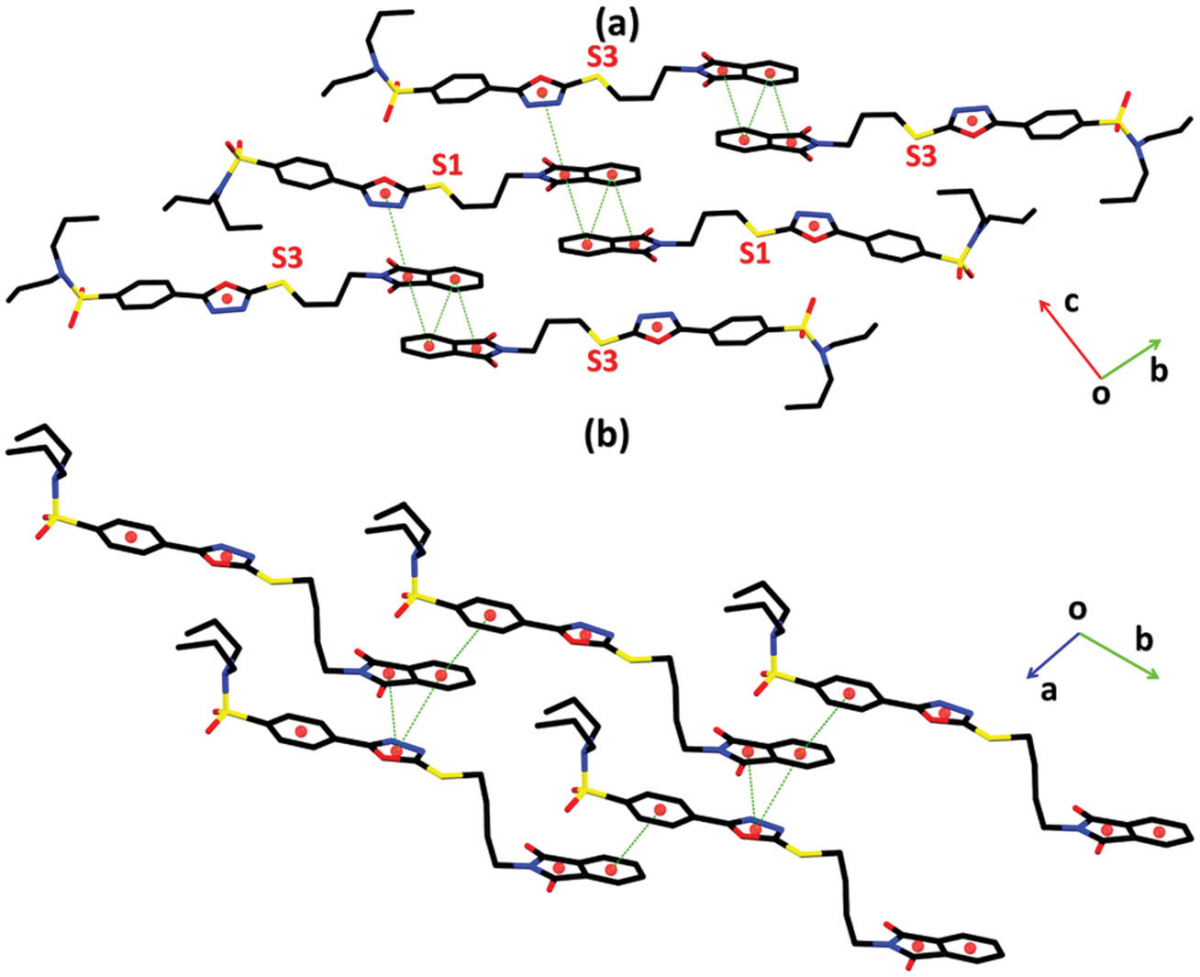
Offset π⋯π stacking interaction of hybrids **(a) 1** and **(b) 2**. Hydrogen atoms are not shown. The major of the disordered propyl groups in hybrid **1** are shown for clarity. Sulphur atoms S1 and S3 are labelled in order to distinguish between molecules **I** and **II** of hybrid **1**.

In hybrid **1**, the molecules of type I are interlinked with molecules of type II through C–H⋯ N and C–H⋯ O bonding (Figure 3(a), Table 2). The molecules of same kind are connected with each other through C–H⋯ O bonding. Due to CH⋯ O bonding, $R_{2}^{2}$(14) H-bonded loop is formed followed by two bifurcated $R_{2}^{2}$(10) loops that are formed by the combination of C–H⋯ O and C-H⋯ N bonding. In hybrid **2**, the molecules are interlinked through C–H⋯ O bonding (Figure 3(b)). The N-atoms of heterocyclic five-membered ring do not involve any type of H-bonding this time. C12 infinite chain of molecules is formed through the combination of C6–H6⋯ O4 and C24–H24A⋯ O1 bonding that runs along the crystallographic *c*-axis. The crystal packing of the hybrid **1** is further stabilised by offset π⋯π stacking interactions between the molecules of similar kind and molecules of an opposite kind with inter-centroid separation ranges from 3.6488(14) to 4.0974(14) Å, and ring offset ranges from 1.077 to 1.279 Å (Figure 4(a), Table 3). Similarly, the crystal packing of the hybrid **2** is further stabilised by offset π⋯π stacking interactions between the symmetry-related molecules with inter-centroid separation ranges from 3.576(3) to 4.039(3) Å and ring offset ranges from 1.037 to 2.248 Å (Figure 4(b), Table 3).

**Table 2.** Hydrogen-bond geometry (Å, °) for hybrids **1, 2**.

	*D—H···A*	*D—H (Å)*	*H···A (Å)*	*D···A (Å)*	*<D—H···A (°)*
***1***	C3—H3···N6^i^	0.95	2.58	3.406 (3)	145
	C28—H28···N2^i^	0.95	2.56	3.393 (3)	147
	C11—H11*A*···O7^i^	0.99	2.59	3.309 (3)	129
	C20*A*—H20*A*···O5	0.99	2.35	2.879 (8)	112
	C20*A*—H20*B*···O10^ii^	0.99	2.65	3.568 (7)	155
	C36—H36*A*···O1^i^	0.99	2.59	3.286 (3)	128
	C41—H41···O10^ii^	0.95	2.61	3.407 (4)	142
	C45*A*—H45*A*···O5^iii^	0.99	2.55	3.472 (6)	154
	C45*A*—H45*B*···O10	0.99	2.38	2.889 (7)	111
***2***	C6—H6···O4^iv^	0.95	2.39	3.177 (6)	140
	C24—H24*A*···O1^v^	0.99	2.58	3.179 (7)	119

**Symmetry codes:** (i) −*x* + 2, −*y* + 2, −*z*; (ii) −*x* + 1, −*y*, −*z* + 1; (iii) −*x* + 2, −*y*, −*z* + 1; (iv) −*x* + 1, −*y* + 1, −*z*; (v) −*x* + 1, −*y* + 1, −*z* + 1.

**Table 3.** The important parameters of offset π⋯π stacking interactions in hybrids **1** and **2**.

	Cg(i)-Cg(j)	D(ij) (Å)	α (°)	Ring offset (Å)
**1**	Cg1-Cg6^i^	4.0974 (14)	52.73 (14)	–
	Cg2-Cg3^ii^	3.7628 (15)	0.89 (13)	1.187
	Cg2-Cg5^iii^	4.0592 (15)	53.01 (14)	–
	Cg3-Cg3^iv^	3.7833 (14)	0.02 (12)	1.243
	Cg6-Cg7^v^	3.7085 (14)	1.34 (13)	1.279
	Cg7-Cg7^v^	3.6488 (14)	0.03 (11)	1.077
**2**	Cg1-Cg2^vi^	3.576 (3)	2.4 (3)	1.037
	Cg1-Cg3^vi^	3.747 (3)	3.8 (3)	1.241
	Cg3-Cg4	4.039 (3)	6.3 (3)	2.248

D(ij) and *α* are the inter-centroid separation and dihedral angles between planes of the interacting rings, respectively.

**Symmetry codes:** (i) *x*, *y*, *z*; (ii) −*x* + 2, −*y* + 3, −*z; (iii) x* + 1, *y*, *z; (iv)* −*x* + 1, −*y* + 2, −*z; (v) x*, −*y* + 1, *z; (vi) x* + 1, −*y* + 1, *z.* For hybrid **1**, Cg1, Cg2, Cg3, Cg5, Cg6 and Cg7 are centroid of (C12/C13/N2/N3/O3), (C1/C2/C7/C8/N1), (C2-C7), (C37/C38/N6/N7/O8), (C26/C27/C32/C33/N5) and (C27-C32) rings, respectively. For hybrid **2**, Cg1, Cg2, Cg3 and Cg4 are centroid of (C13/C14/N2/N3/O3), (C1/C2/C7/C8/N1), (C2-C7) and (C15-C20) rings, respectively.

### 3.3. Molecular electrostatic potential (MEP) calculations

**Figure 5.**
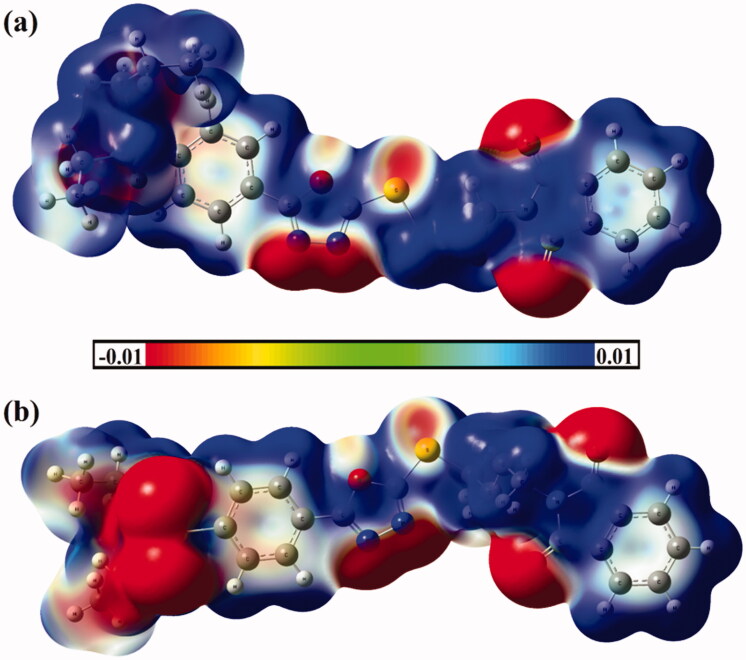
Molecular electrostatic potential (MEP) maps of hybrids (a) **1** and (b) **2** are plotted onto 0.002 au electron density contours. The electrostatic potential varies from −0.01 (red) to +0.01 (blue) au.

Molecular electrostatic potential (MEP) analysis has been recently apprehended as an informative method to conceive the nature of the chemical systems by indicating the electrophilic and nucleophilic sites on their molecular surfaces^74^. The investigated hybrids **1** and **2** are first optimised. No imaginary frequency is noticed for the studied hybrids, ensuring that the obtained geometries are true minima. On the optimised monomers, MEP maps are generated using 0.002 au electron density contours. The generated MEP maps of hybrids **1** and **2** are displayed in Figure 5.

Based on the data shown in Figure 5, apparent nucleophilic nature is detected with the obvious red region along the molecular surface of the sulphonamide, nitrogens of oxadiazole, and oxygens of phthalimide moieties. In contrast, the electrophilic sites are noticed along the rest molecular surface of the investigated hybrids **1** and **2** that are noticed by the occurrence of deep blue colour regions.

### 3.4. Frontier molecular orbitals (FMOs) analysis

**Figure 6.**
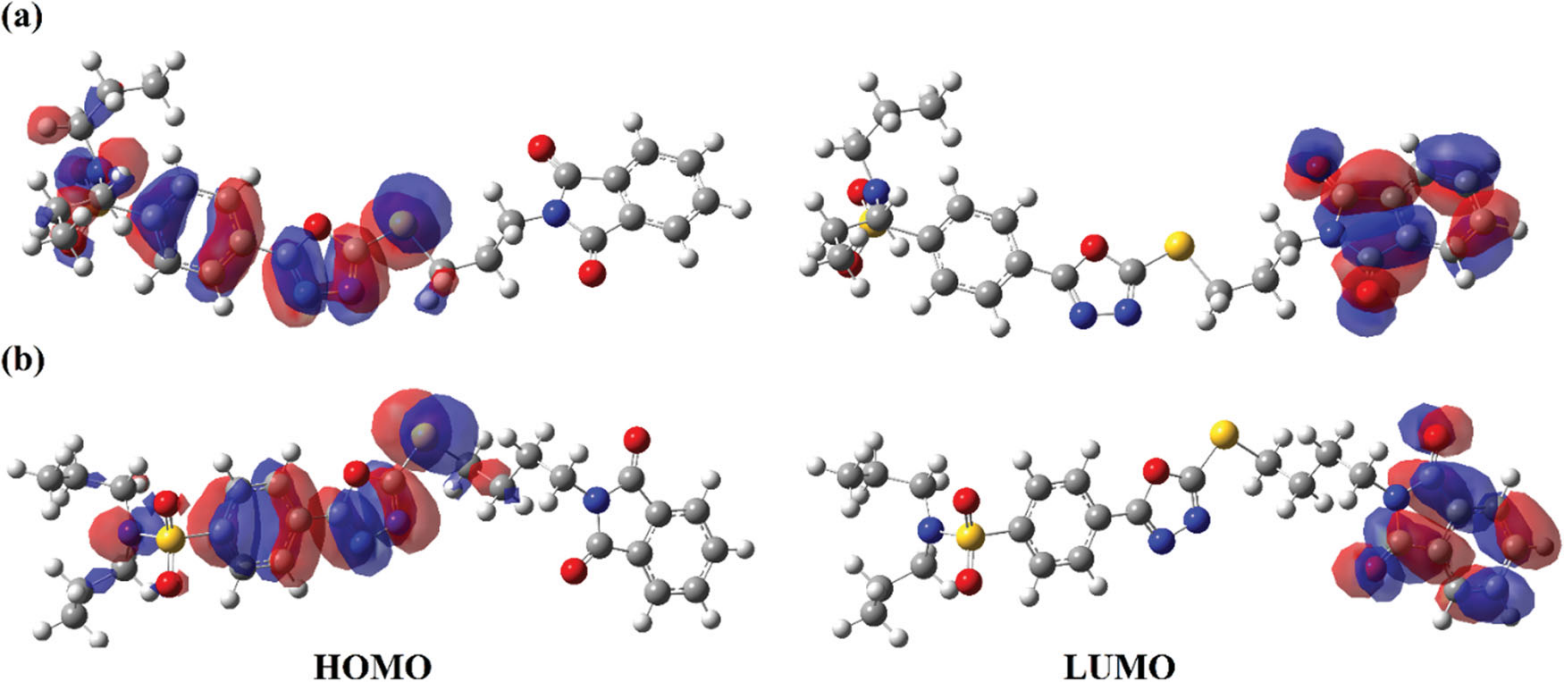
Frontier molecular orbitals (FMOs), including the highest occupied molecular orbitals (HOMO) and lowest unoccupied molecular orbitals (LUMO) of hybrids (a) **1** and (b) **2**.

Several studies have addressed frontier molecular orbitals (FMOs) analysis as a consistent tool to elucidate the convenient sites on the molecular surfaces for inter- and intra-interactions. Using FMOs, the highest occupied molecular orbitals (HOMO) and lowest unoccupied molecular orbitals (LUMO) are generated to assess the stability of the studied hybrids and are illustrated in Figure 6.

Subject to data graphed in Figure 6, the HOMO orbitals are apparently delocalised on the oxadiazole and benzenesulfonamide moieties. While the dispersion of isodensities in LUMOs of the investigated hybrids is notably concentrated on the phthalimide moiety, outlining the occurrence of charge transfer from oxadiazole and benzenesulfonamide to the phthalimide moiety.

### 3.5. QTAIM and NCI analyses

**Figure 7.**
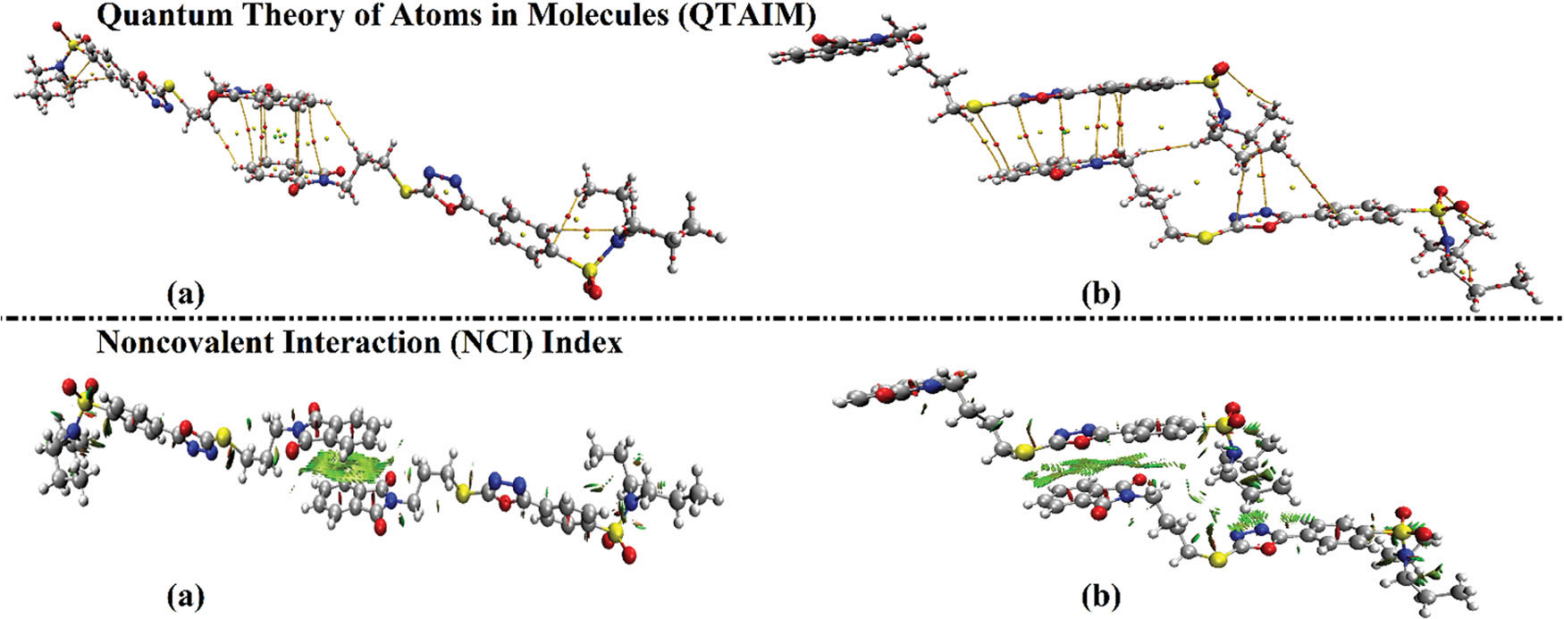
Quantum theory of atoms in molecules (QTAIM) and 3 D noncovalent interaction (NCI) isosurfaces of hybrids (a) **1** and (b) **2**. The isosurfaces are generated with a reduced density gradient value of 0.50 au and coloured from blue to red according to sign(λ_2_)*ρ* ranging from −0.035 (blue) to 0.020 (red) au.

Quantum theory of atoms in molecules (QTAIM) protocol^53^ and noncovalent interaction (NCI) index^54^ are utilised to elucidate the inter- and intra-molecular interactions within the dimers of hybrids **1** and **2**. In the context of QTAIM, the bond paths (BPs) and bond critical points (BCPs) are generated and are depicted in Figure 7. Through NCI analysis, the three-dimensional (3 D) colour-mapped isosurfaces of inter- and intra-molecular interaction regions are extracted and are graphed in Figure 7.

According to the results presented in Figure 7, the occurrence of intra- and inter-molecular contacts within the inspected hybrids are conspicuously unveiled by the existence of BPs and BCPs within both examined dimers, particularly π···π stacking interactions. In line with QTAIM affirmations, obvious green isosurfaces re apparently noticed through the generated 3 D NCI plots.

### 3.6. Hirshfeld surface (HS) analysis

**Figure 8.**
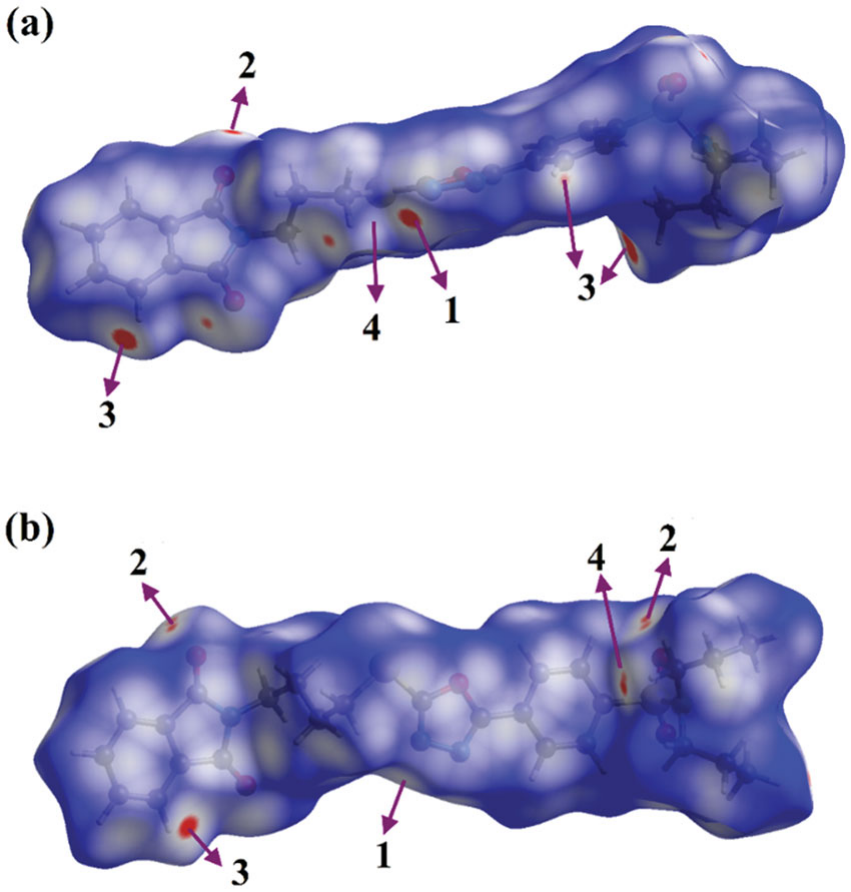
View of the Hirshfeld surfaces mapped over *d*_norm_ property of (a) hybrid **1** and (b) hybrid **2**. The labels 1, 2, 3, and 4 represent N···H/H···N, O···H/H···O, C···H/H···C, and S···H/H···S interactions, respectively.

**Figure 9.**
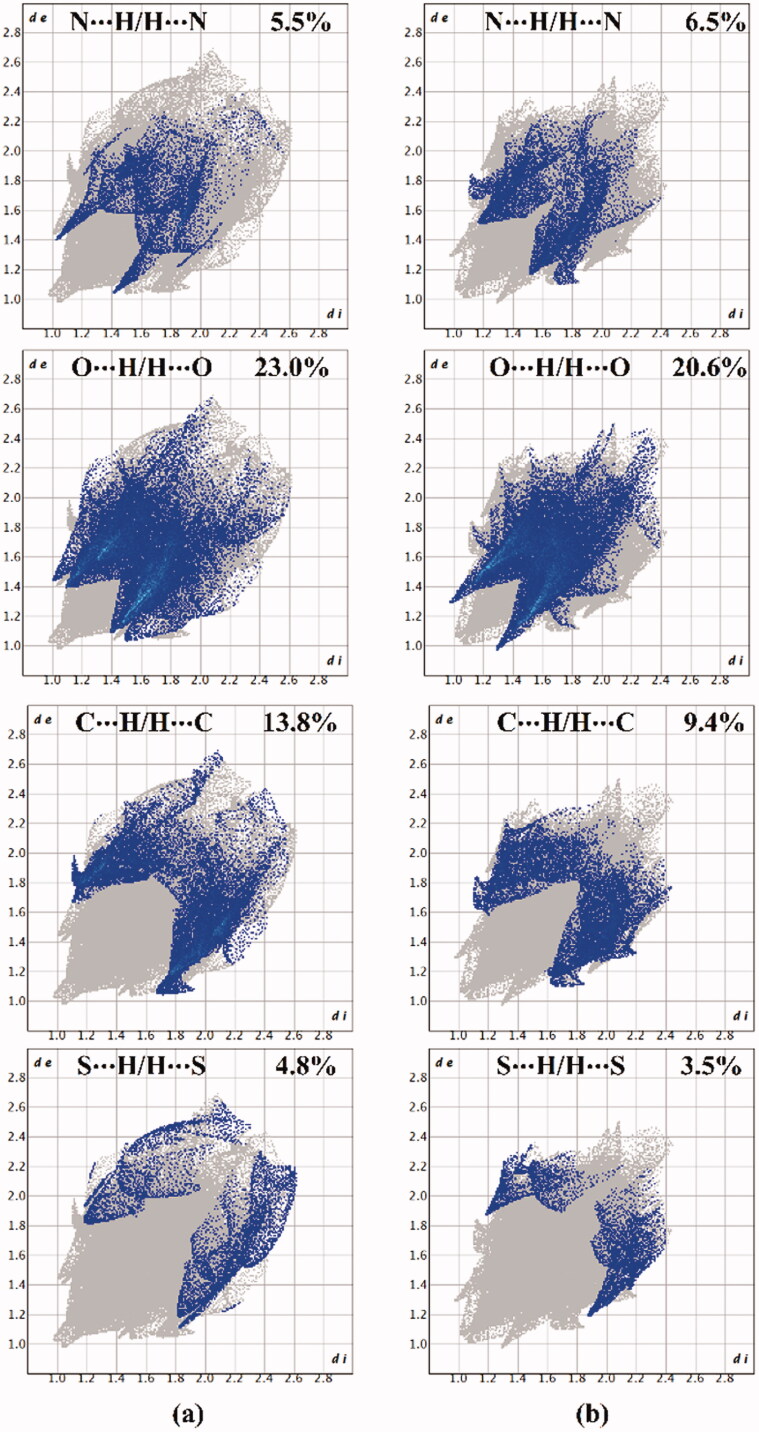
2 D fingerprint plots of hybrids (a) **1** and (b) **2**.

**Figure 10.**
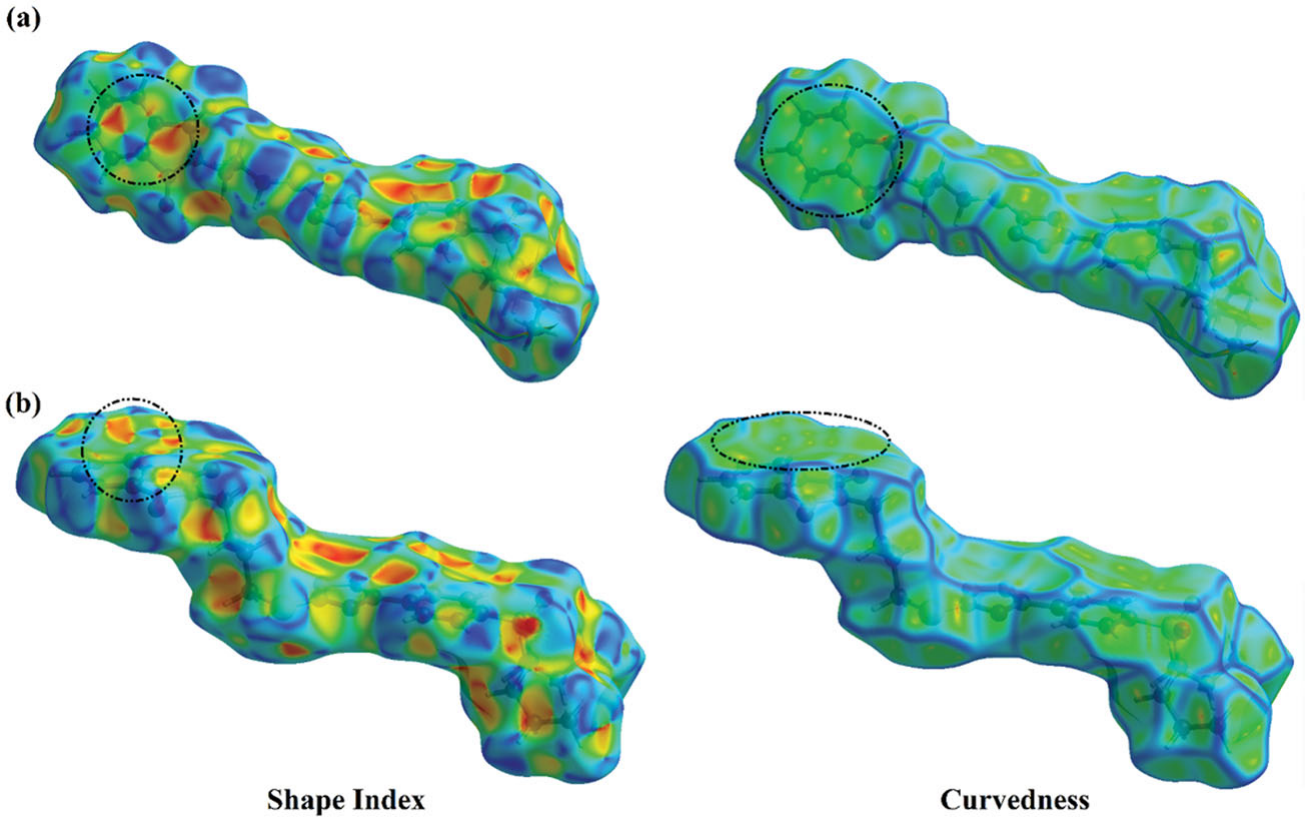
Hirshfeld surfaces of hybrids (a) **1** and (b) **2** mapped over Shape index and Curvedness properties.

Hirshfeld surface (HS) analysis is documented as an authoritative tool to qualitatively assess the nature of intermolecular interactions within crystal structures and thoroughly identify the interactions throughout the surface around the molecules^75–80^. Hirshfeld surfaces, including the *d*_norm_ along with its associated 2 D fingerprints, shape index, and curvedness, are mapped for the investigated hybrids **1** and **2**. Figures 8 and 9 illustrate the *d*_norm_ maps and their associated 2 D fingerprint plots, respectively. The generated shape index and curvedness maps are given in Figure 10.

As shown in Figure 8, the H···N/N···H contacts are detected with obvious large red regions labelled 1, which are common in the *d*_norm_ maps of both hybrids. Such contacts are also noticed in the 2 D fingerprint plots as a pair of symmetrical spikes at (*d*_e_ + *d*_i_) ∼ 2.5 Å and 3.0 Å for hybrids **1** and **2**, respectively (Figure 9). The H···N/N···H contacts exhibit 5.5% and 6.5% of the total Hirshfeld surface area of hybrids **1** and **2**, respectively.

The existence of red regions with label 2 in the Hirshfeld surfaces of two hybrids could be ascribed to the occurrence of the reciprocal H···O and O···H contacts that were found in the 2 D fingerprint plots at (*d*_e_ + *d*_i_) ∼ 3.5 Å and 2.3 Å for hybrids **1** and **2**, respectively. Turning to the proportions of H···C/C···H contacts, prominent red regions are noticed with 13.8% and 9.4% of the total Hirshfeld surface area and characterised by spikes at (*d*_e_ + *d*_i_) ∼ 3.0 Å and 4.1 Å for hybrids **1** and **2**, respectively. The H···S/S···H contacts attributed to weak C–H···S interactions are observed with label 4, as white and red regions in the *d*_norm_ maps of hybrids **1** and **2** with 4.8% and 3.5% contributions, respectively. Conspicuously, the π···π stacking interactions show variant contributions to the total Hirshfeld surfaces of hybrids **1** and **2** with values of 2.7% and 3.8%, respectively.

Crucially, the occurrence of the π···π stacking interactions is obviously well-characterised in the Hirshfeld surfaces mapped over Shape index and Curvedness properties by the existence of the complementary pair of red and blue triangles in Shape index and the flat green area in Curvedness (Figure 10).

### 3.7. α-Amylase inhibition activity

*α*-Amylase is an oligosaccharide endoglycosidase that is found in the body fluids of humans and secreted by the pancreas. Its function is to break down the *α*-bonds of large *α*-linked starch and glycogen into glucose and maltose. Uncontrolled activity of *α*-amylase results in excessive accumulation of blood glucose responsible for hyperglycaemia. *α*-Amylase inhibitors decrease the activity of the enzyme and hence the level of glucose in the blood. *α*-Amylase can be used to control hyperglycaemia by decreasing the activity of the *α*-amylase.

**Figure 11.**
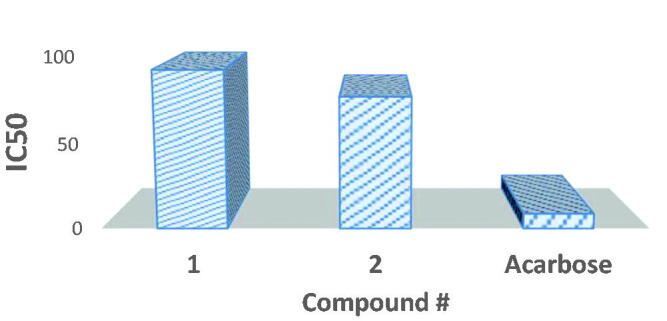
Graphical representation of α-amylase inhibition of probenecid derived two *S*-alkylphthalimide-oxadiazole-benzenesulfonamide hybrids (**1** and **2**) at different concentrations.

The inhibitory potential of synthesised probenecid-derived *S*-alkylphthalimide-oxadiazole-benzenesulfonamide hybrids (**1** and **2**) is evaluated against *α*-amylase. Both hybrids (**1** and **2)** show potent inhibitory potential. The potent inhibitory potential is tested at concentration levels 10, 100, 150, and 200 μg/mL where hybrids **1** and **2** delivered 77.37% and 83.36% inhibition of the *α*-amylase at 200 μg/mL, respectively (Table 4). A logarithmic regression curve is established with the help of percent inhibition potential (% I) at four different concentrations for each hybrid to determine the concentration of inhibition at 50% of the *α*-amylase activity (IC_50_ μg/mL). Acarbose, a commercially established *α*-amylase inhibitor, is used as standard (inhibitory activity with 82.55% inhibition at 200 μg/mL concentration and IC_50_ value of 8.80 ± 0.21 μg/mL). Both hybrids **1** and **2** have shown IC_50_ values of 92.23 ± 0.23 and 76.92 ± 0.19 μg/mL, respectively (graphical evaluation is displayed in Figure 11).

**Table 4.** *α*-Amylase inhibition values of probenecid derived two *S*-alkylphthalimide-oxadiazole-benzenesulfonamide hybrids (**1** and **2**).

Compound	Concentration*(μ*g/mL)	% of inhibition	IC_50_ value (*μ*g/mL)
**1**	10	20.24	92.23 ± 0.23
	50	43.68	
	100	58.11	
	150	64.59	
	200	77.37	
**2**	10	33.19	76.92 ± 0.19
	50	42.54	
	100	52.21	
	150	72.95	
	200	83.36	
**Acarbose**	10	55.21	8.80 ± 0.21
	100	73.83	
	200	82.55	

Structure-activity relationship of hybrids **1** and **2** discloses that hybrid **2,** with four carbon bridge between oxadiazole and phthalimide moieties, is a more active inhibitor against *α*-amylase inhibition than hybrid **1,** which contains a three-carbon bridge.

### 3.8. Molecular docking

**Figure 12.**
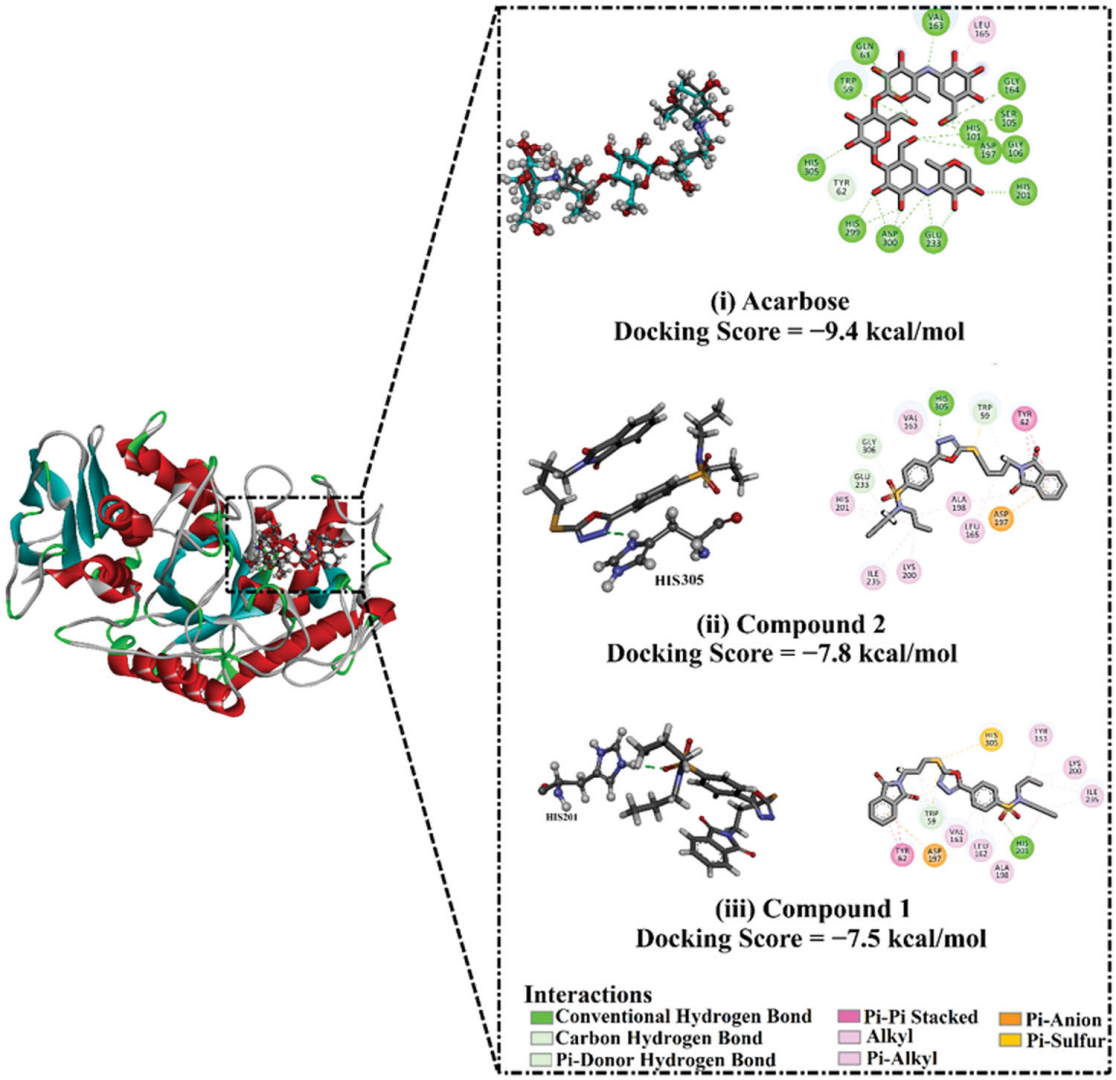
(i) 3D and 2D representations of the anticipated docking pose (in cyan) and experimental structure (in gray) of acarbose and the predicted binding modes of compounds (ii) **2**, and (iii) **1** with *α*-amylase.

Prior to molecular docking calculations, the reliability of an *in silico* approach for anticipating the binding mode of the *α*-amylase ligand is inspected. The co-crystallised acarbose ligand is redocked towards the *α*-amylase, in addition to the expected docking pose is compared to the resolved structure (PDB ID: 1OSE^65^) (Figure 12). As shown in Figure 12, the expected docking pose is extremely comparable to the native structure, with a binding affinity of −9.4 kcal/mol and an RMSD of 0.23 Å. This data comparison discloses the outperformance of the AutoDock4.2.6 software to portend the resolved binding mode of *α*-amylase ligand. The robust binding of acarbose with *α*-amylase is ascribed to its capacity to form twenty hydrogen bonds with GLU233, ASP300, HIS299, HIS305, ASP197, HIS101, TRP59, GLN63, GLY164, VAL163, SER105, GLY106, and HIS201 with bond lengths ranging from 1.61 to 3.28 Å (Figure 12).

Molecular docking calculations show that compound **2** demonstrates a satisfactory binding affinity against *α*-amylase with a docking score of −7.8 kcal/mol (Figure 12). Compound **1** displays a good binding affinity with a docking score of −7.5 kcal/mol (Figure 12). The good potentialities of compounds **1** and **2** as *α*-amylase inhibitors are imputed to their capability to exhibit a hydrogen bond, hydrophobic, van der Waals interactions, and pi-based interactions with the proximal amino acids inside the binding pocket of *α*-amylase (Figure 12).

## 4. Conclusions

In conclusion, probenecid-derived two *S*-alkylphthalimide-oxadiazole-benzenesulfonamide hybrids (**1** and **2**) are accessed in very good yields. The ultimate molecular structures of both compounds are analysed through FT-IR, ^1^HNMR, ^13^CNMR and are validated with the help of X-ray diffraction analysis. MEP maps conspicuously announce both the nucleophilic and electrophilic nature of the studied hybrids. The existence of the N···H/H···N, O···H/H···O, C···H/H···C, and S···H/H···S contacts is unequivocally unveiled within the generated Hirshfeld surfaces. *α-*Amylase inhibition studies have shown that hybrids **1** and **2** have good enzyme inhibition potential against the *α-*amylase. The synthesised compounds are *in silico* investigated towards *α-*amylase with the assistance of the molecular docking technique. The binding affinities reveal that compounds **1** and **2** are potent *α-*amylase inhibitors.

## References

[1] Bostrom J, Hogner A, Llinas A, et al. Oxadiazoles in medicinal chemistry. J Med Chem 2012;55:1464–30. 10.1021/jm2013248 22185670

[2] Pace A, Pierro P. The new era of 1,2,4-oxadiazoles. Org Biomol Chem 2009;7:4337–48. 19830279 10.1039/b908937c

[3] Holla BS, Gonsalves R, Shenoy S. Synthesis and antibacterial studies of a new series of 1,2-bis(1,3, 4-oxadiazol-2-yl)ethanes and 1,2-bis(4-amino-1,2, 4-triazol-3-yl)ethanes. Eur J Med Chem 2000;35:267–71. 10758288 10.1016/s0223-5234(00)00154-9

[4] Jones AM, Helm JM. Emerging treatments in cystic fibrosis. Drugs 2009;69:1903–10. 19747007 10.2165/11318500-000000000-00000

[5] Ohmoto K, Okuma M, Yamamoto T, et al. Design and synthesis of new orally active inhibitors of human neutrophil elastase. Bioorg Med Chem 2001;9:1307–23. 11377188 10.1016/s0968-0896(01)00007-4

[6] Ono M, Haratake M, Saji H, Nakayama M. Development of novel beta-amyloid probes based on 3,5-diphenyl-1,2,4-oxadiazole. Bioorg Med Chem 2008;16:6867–72. 18550375 10.1016/j.bmc.2008.05.054

[7] Foss FW, Jr., Mathews TP, Kharel Y, et al. Synthesis and biological evaluation of sphingosine kinase substrates as sphingosine-1-phosphate receptor prodrugs. Bioorg Med Chem 2009;17:6123–36. 19632123 10.1016/j.bmc.2009.04.015 PMC2793099

[8] Tarasenko M, Duderin N, Sharonova T, et al. Room-temperature synthesis of pharmaceutically important carboxylic acids bearing the 1,2,4-oxadiazole moiety. Tetrahedron Lett 2017;58:3672–7.

[9] Mohamed NA, Al-afaleq EI. Aromatic 1,3,4-oxadiazoles as thermal stabilizers for rigid poly(vinyl chloride). Polymer 1999;40:617–27.

[10] Casu MB, Imperia P, Schulz B, Schrader S. Electronic structure at the interface between metals and new materials for organic light emitting diodes. Surf Sci 2002;507-510:666–71.

[11] Jones RM, Leonard JN, Buzard DJ, Lehmann J. GPR119 agonists for the treatment of type 2 diabetes. Expert Opin Ther Pat 2009;19:1339–59. 19780700 10.1517/13543770903153878

[12] Ducharme Y, Blouin M, Brideau C, et al. The discovery of setileuton, a potent and selective 5-lipoxygenase inhibitor. ACS Med Chem Lett 2010;1:170–4. 24900191 10.1021/ml100029k PMC4007958

[13] Unangst PC, Shrum GP, Connor DT, et al. Novel 1,2,4-oxadiazoles and 1,2,4-thiadiazoles as dual 5-lipoxygenase and cyclooxygenase inhibitors. J Med Chem 1992;35:3691–8. 1433181 10.1021/jm00098a015

[14] Cottrell DM, Capers J, Salem MM, et al. Antikinetoplastid activity of 3-aryl-5-thiocyanatomethyl-1,2,4-oxadiazoles. Bioorg Med Chem 2004;12:2815–24. 15142541 10.1016/j.bmc.2004.03.054

[15] Ohmoto K, Yamamoto T, Horiuchi T, et al. Design and synthesis of new orally active nonpeptidic inhibitors of human neutrophil elastase. J Med Chem 2000;43:4927–9. 11150162 10.1021/jm0004087

[16] Hamdani SS, Khan BA, Ahmed MN, et al. Synthesis, crystal structures, computational studies and α-amylase inhibition of three novel 1,3,4-oxadiazole derivatives. J Mol Struct 2020;1200:127085.

[17] Khan BA, Zafar S, Mughal EU, et al. Design and synthesis of novel 1,3,4-oxadiazole derivatives bearing azo moiety as biologically significant scaffolds. Lett Drug Des Discov 2018;15:1346–55.

[18] Du Y, Zhuang J, Liu H, et al. Tuning the band gap in silicene by oxidation. ACS Nano 2014;8:10019–25. 25248135 10.1021/nn504451t

[19] Fish PV, Allan GA, Bailey S, et al. Potent and selective nonpeptidic inhibitors of procollagen C-proteinase. J Med Chem 2007;50:3442–56. 17591762 10.1021/jm061010z

[20] Zhang HZ, Kasibhatla S, Kuemmerle J, et al. Discovery and structure-activity relationship of 3-aryl-5-aryl-1,2,4-oxadiazoles as a new series of apoptosis inducers and potential anticancer agents. J Med Chem 2005;48:5215–23. 16078840 10.1021/jm050292k

[21] Xu J, Wei L, Mathvink RJ, et al. Discovery of potent, selective, and orally bioavailable oxadiazole-based dipeptidyl peptidase IV inhibitors. Bioorg Med Chem Lett 2006;16:5373–7. 16919457 10.1016/j.bmcl.2006.07.061

[22] Harfenist M, Heuser DJ, Joyner CT, et al. Selective inhibitors of monoamine oxidase. 3. Structure-activity relationship of tricyclics bearing imidazoline, oxadiazole, or tetrazole groups. J Med Chem 1996;39:1857–63. 8627609 10.1021/jm950595m

[23] Shen HC, Ding FX, Raghavan S, et al. Discovery of a biaryl cyclohexene carboxylic acid (MK-6892): a potent and selective high affinity niacin receptor full agonist with reduced flushing profiles in animals as a preclinical candidate. J Med Chem 2010;53:2666–70. 20184326 10.1021/jm100022r

[24] Oliveira VS, Pimenteira C, da Silva-Alves DC, et al. The enzyme 3-hydroxykynurenine transaminase as potential target for 1,2,4-oxadiazoles with larvicide activity against the dengue vector Aedes aegypti. Bioorg Med Chem 2013;21:6996–7003. 24095017 10.1016/j.bmc.2013.09.020

[25] Sangshetti JN, Chabukswar AR, Shinde DB. Microwave assisted one pot synthesis of some novel 2,5-disubstituted 1,3,4-oxadiazoles as antifungal agents. Bioorg Med Chem Lett 2011;21:444–8. 21095127 10.1016/j.bmcl.2010.10.120

[26] Ramsbeck D, Buchholz M, Koch B, et al. Structure-activity relationships of benzimidazole-based glutaminyl cyclase inhibitors featuring a heteroaryl scaffold. J Med Chem 2013;56:6613–25. 23886302 10.1021/jm4001709

[27] Cunningham RF, Israili ZH, Dayton PG. Clinical pharmacokinetics of probenecid. Clin Pharmacokinet 1981;6:135–51. 7011657 10.2165/00003088-198106020-00004

[28] Mason RM. Studies on the effect of probenecid (benemid) in gout. Ann Rheum Dis 1954;13:120–30. 13171805 10.1136/ard.13.2.120 PMC1030399

[29] Mollica A, Costante R, Akdemir A, et al. Exploring new probenecid-based carbonic anhydrase inhibitors: synthesis, biological evaluation and docking studies. Bioorg Med Chem 2015;23:5311–8. 26264840 10.1016/j.bmc.2015.07.066

[30] World Health Organization. Global diffusion of eHealth: making universal healthcoverage achievable: report of the third global survey on eHealth. Geneva: World Health Organization; 2017.

[31] World Health Organization. (2021). Diabetes.

[32] Chobanian AV, Bakris GL, Black HR, et al. The seventh report of the joint national committee on prevention, detection, evaluation, and treatment of high blood pressure: the JNC 7 report. JAMA 2003;289:2560–72. 12748199 10.1001/jama.289.19.2560

[33] Gani RS, Kudva AK, Timanagouda K, et al. Synthesis of novel 5-(2,5-bis(2,2,2-trifluoroethoxy)phenyl)-1,3,4-oxadiazole-2-thiol derivatives as potential glucosidase inhibitors. Bioorg Chem 2021;114:105046. 34126575 10.1016/j.bioorg.2021.105046

[34] Sipos A, Szennyes E, Hajnal NE, et al. Dual-target compounds against type 2 diabetes mellitus: proof of concept for sodium dependent glucose transporter (SGLT) and glycogen phosphorylase (GP) inhibitors. Pharmaceuticals (Basel) 2021;14:364. 33920838 10.3390/ph14040364 PMC8071193

[35] Siwach A, Verma PK. Therapeutic potential of oxadiazole or furadiazole containing compounds. BMC Chem 2020;14:70. 33372629 10.1186/s13065-020-00721-2 PMC7722446

[36] Lim KS, Cho JY, Kim BH, et al. Pharmacokinetics and pharmacodynamics of LC15-0444, a novel dipeptidyl peptidase IV inhibitor, after multiple dosing in healthy volunteers. Br J Clin Pharmacol 2009;68:883–90. 20002082 10.1111/j.1365-2125.2009.03376.x PMC2810799

[37] Singh P, Jangra PK. Oxadiazoles: a novel class of anti-convulsant agents. Der Chemica Sinica 2010;1:118–23.

[38] Husain A, Ajmal M. Synthesis of novel 1,3,4-oxadiazole derivatives and their biological properties. Acta Pharm 2009;59:223–33. 19564146 10.2478/v10007-009-0011-1

[39] Martínez R, Zamudio GJN, Pretelin-Castillo G, et al. Synthesis and antitubercular activity of new N-[5-(4-chlorophenyl)-1,3,4-oxadiazol-2-yl]-(nitroheteroaryl)carboxamides. Heterocycl Comm 2019;25:52–9.

[40] Arshad M, Jadoon M, Iqbal Z, et al. Synthesis, molecular structure, quantum mechanical studies and urease inhibition assay of two new isatin derived sulfonylhydrazides. J Mol Struct 2017;1133:80–9.

[41] Sherzaman S, Sadiq-ur-Rehman , Ahmed MN, Khan BA, et al. Thiobiuret based Ni(II) and Co(III) complexes: synthesis, molecular structures and DFT studies. J Mol Struct 2017;1148:388–96.

[42] Ahmed MN, Sadiq B, Al-Masoudi NA, et al. Synthesis, crystal structures, computational studies and antimicrobial activity of new designed bis((5-aryl-1,3,4-oxadiazol-2-yl)thio)alkanes. J Mol Struct 2018;1155:403–13.

[43] Ahmed MN, Yasin KA, Hameed S, et al. Synthesis, structural studies and biological activities of three new 2-(pentadecylthio)-5-aryl-1,3,4-oxadiazoles. J Mol Struct 2017;1129:50–9.

[44] Ahmed MN, Ashraf I, Yasin KA, et al. Synthesis, characterization, anti-leishmanial activity and in silico studies of 5-(4-methoxyphenyl)-2-(undecylthio)-1,3,4-oxadiazole. J Chem Soc Pak 2018;40:773–81.

[45] Sheldrick GM. SHELXT - integrated space-group and crystal-structure determination. Acta Crystallogr A Found Adv 2015;71:3–8. 25537383 10.1107/S2053273314026370 PMC4283466

[46] Sheldrick GM. Crystal structure refinement with SHELXL. Acta Crystallogr C Struct Chem 2015;71:3–8. 25567568 10.1107/S2053229614024218 PMC4294323

[47] Farrugia LJ. WinGX and ORTEP for Windows: an update. J Appl Crystallogr 2012;45:849–54.

[48] Spek AL. Structure validation in chemical crystallography. Acta Crystallogr D Biol Crystallogr 2009;65:148–55. 19171970 10.1107/S090744490804362X PMC2631630

[49] Macrae CF, Sovago I, Cottrell SJ, et al. Mercury 4.0: from visualization to analysis, design and prediction. J Appl Crystallogr 2020;53:226–35. 32047413 10.1107/S1600576719014092 PMC6998782

[50] Becke AD. Density-functional exchange-energy approximation with correct asymptotic behavior. Phys Rev A Gen Phys 1988;38:3098–100. 9900728 10.1103/physreva.38.3098

[51] Lee C, Yang W, Parr RG. Development of the Colle-Salvetti correlation-energy formula into a functional of the electron density. Phys Rev B Condens Matter 1988;37:785–9. 9944570 10.1103/physrevb.37.785

[52] Ibrahim MAA. Molecular mechanical perspective on halogen bonding. J Mol Model 2012;18:4625–38. 22643975 10.1007/s00894-012-1454-8

[53] Bader RFW. Atoms in molecules. Acc Chem Res 1985;18:9–15.

[54] Johnson ER, Keinan S, Mori-Sanchez P, et al. Revealing noncovalent interactions. J Am Chem Soc 2010;132:6498–506. 20394428 10.1021/ja100936w PMC2864795

[55] Lu T, Chen F. Multiwfn: a multifunctional wavefunction analyzer. J Comput Chem 2012;33:580–92. 22162017 10.1002/jcc.22885

[56] Humphrey W, Dalke A, Schulten K. VMD: visual molecular dynamics. J Mol Graph 1996;14:33–8. 8744570 10.1016/0263-7855(96)00018-5

[57] Frisch J, Trucks GW, Schlegel HB, et al., Gaussian, Inc., Wallingford CT, Gaussian 09, Revision E.01. 2009.

[58] Spackman MA, Jayatilaka D. Hirshfeld surface analysis. CrystEngComm 2009;11:19–32.

[59] Turner M, McKinnon J, Wolff S, et al. CrystalExplorer17. The University of Western Australia, Australia; 2017.

[60] Ibrahim MAA, Abdeljawaad KAA, Abdelrahman AHM, et al. Non-beta-lactam allosteric inhibitors target methicillin-resistant *Staphylococcus aureus*: an in silico drug discovery study. Antibiotics (Basel) 2021;10:934. 34438984 10.3390/antibiotics10080934 PMC8388891

[61] Ibrahim MAA, Abdelrahman AHM, Atia MAM, et al. Blue biotechnology: computational screening of sarcophyton cembranoid diterpenes for SARS-CoV-2 main protease inhibition. Mar Drugs 2021;19:391. 34356816 10.3390/md19070391 PMC8308023

[62] Ibrahim MAA, Abdelrahman AHM, Hegazy MF. In-silico drug repurposing and molecular dynamics puzzled out potential SARS-CoV-2 main protease inhibitors. J Biomol Struct Dyn 2021;39:5756–67. 32684114 10.1080/07391102.2020.1791958 PMC7441803

[63] Ibrahim MAA, Abdelrahman AHM, Mohamed TA, et al. In silico mining of terpenes from red-sea invertebrates for SARS-CoV-2 main protease (m(pro)) inhibitors. Molecules 2021;26:2082. 33916461 10.3390/molecules26072082 PMC8038614

[64] Ibrahim MAA, Badr EAA, Abdelrahman AHM, et al. In Silico targeting human multidrug transporter ABCG2 in breast cancer: database screening, molecular docking, and molecular dynamics study. Mol Inform 2022;41:e2060039. 34491628 10.1002/minf.202060039

[65] Gilles C, Astier JP, Marchis-Mouren G, et al. Crystal structure of pig pancreatic alpha-amylase isoenzyme II, in complex with the carbohydrate inhibitor acarbose. Eur J Biochem 1996;238:561–9. 8681972 10.1111/j.1432-1033.1996.0561z.x

[66] Gordon JC, Myers JB, Folta T, et al. H++: a server for estimating pKas and adding missing hydrogens to macromolecules. Nucleic Acids Res 2005;33:W368–371. 15980491 10.1093/nar/gki464 PMC1160225

[67] OMEGA 2.5.1.4; OpenEye Scientific Software: Santa Fe, NM; 2013.

[68] Hawkins PC, Skillman AG, Warren GL, et al. Conformer generation with OMEGA: algorithm and validation using high quality structures from the Protein Databank and Cambridge Structural Database. J Chem Inf Model 2010;50:572–84. 20235588 10.1021/ci100031x PMC2859685

[69] Halgren TA. MMFF VI. MMFF94s option for energy minimization studies. J Comput Chem 1999;20:720–9. 34376030 10.1002/(SICI)1096-987X(199905)20:7<720::AID-JCC7>3.0.CO;2-X

[70] *SZYBKI* 1.9.0.3; OpenEye Scientific Software: Santa Fe (NM); 2016.

[71] Morris GM, Huey R, Lindstrom W, et al. AutoDock4 and AutoDockTools4: automated docking with selective receptor flexibility. J Comput Chem 2009;30:2785–91. 19399780 10.1002/jcc.21256 PMC2760638

[72] Forli S, Huey R, Pique ME, et al. Computational protein-ligand docking and virtual drug screening with the AutoDock suite. Nat Protoc 2016;11:905–19. 27077332 10.1038/nprot.2016.051 PMC4868550

[73] Mansourian M, Fassihi A, Saghaie L, et al. QSAR and docking analysis of A2B adenosine receptor antagonists based on non-xanthine scaffold. Med Chem Res 2015;24:394–407.

[74] Weiner PK, Langridge R, Blaney JM, et al. Electrostatic potential molecular surfaces. Proc Natl Acad Sci U S A 1982;79:3754–8. 6285364 10.1073/pnas.79.12.3754 PMC346505

[75] Anitha K, Sivakumar S, Arulraj R, et al. Synthesis, crystal structure, DFT calculations and Hirshfeld surface analysis of 3-butyl-2,6-bis-(4-fluoro-phen-yl)piperidin-4-one. Acta Crystallogr E Crystallogr Commun 2020;76:651–5. 32431926 10.1107/S2056989020004636 PMC7199252

[76] Arulraj R, Sivakumar S, Kaur M, et al. Crystal structures of three 3-chloro-3-methyl-2,6-di-aryl-piperidin-4-ones. Acta Crystallogr E Crystallogr Commun 2017;73:107–11. 28217321 10.1107/S2056989016020661 PMC5290544

[77] McKinnon JJ, Spackman MA, Mitchell AS. Novel tools for visualizing and exploring intermolecular interactions in molecular crystals. Acta Crystallogr B 2004;60:627–68. 15534375 10.1107/S0108768104020300

[78] McKinnon JJ, Jayatilaka D, Spackman MA. Towards quantitative analysis of intermolecular interactions with Hirshfeld surfaces. ChemComm 2007;37:3814–6. 10.1039/b704980c 18217656

[79] Haroon M, Akhtar T, Yousuf M, et al. Synthesis, crystal structure, Hirshfeld surface investigation and comparative DFT studies of ethyl 2-[2-(2-nitrobenzylidene)hydrazinyl]thiazole-4-carboxylate. BMC Chem 2022;16:18. 35317817 10.1186/s13065-022-00805-1 PMC8941777

[80] Ashfaq M, Ali A, Tahir MN, et al. Synthesis, single-crystal exploration, hirshfeld surface analysis, and DFT investigation of the thiosemicarbazones. J Mol Struct 2022;1262:133088.

